# Crystal Structure, Luminescence and Electrical Conductivity of Pure and Mg^2+^-Doped β-Ga_2_O_3_-In_2_O_3_ Solid Solutions Synthesized in Oxygen or Argon Atmospheres

**DOI:** 10.3390/ma17061391

**Published:** 2024-03-18

**Authors:** Andriy Luchechko, Vyacheslav Vasyltsiv, Markiyan Kushlyk, Vasyl Hreb, Dmytro Slobodzyan, Leonid Vasylechko, Yaroslav Zhydachevskyy

**Affiliations:** 1 Department of Sensor and Semiconductor Electronics, Ivan Franko National University of Lviv, Tarnavskogo Str., 107, 79017 Lviv, Ukraine; 2 Department of Semiconductor Electronics, Lviv Polytechnic National University, S. Bandera Str., 12, 79013 Lviv, Ukraine; vasyl.m.hreb@lpnu.ua (V.H.); leonid.o.vasylechko@lpnu.ua (L.V.); 3 Institute of Physics, Polish Academy of Sciences, Aleja Lotników 32/46, 02-668 Warsaw, Poland; 4 Department of Physics and Methods of Teaching Physics, Berdyansk State Pedagogical University, Shmidta Str. 4, 71100 Berdyansk, Ukraine

**Keywords:** monoclinic Ga_2_O_3_-In_2_O_3_, crystal structure, luminescence, electrical conductivity, point defects

## Abstract

Undoped and Mg^2+^-doped β-Ga_2_O_3_-20% In_2_O_3_ solid solution microcrystalline samples were synthesized using the high-temperature solid-state chemical reaction method to investigate the influence of native defects on structural, luminescent, and electrical properties. The synthesis process involved varying the oxygen partial pressure by synthesizing samples in either an oxygen or argon atmosphere. X-ray diffraction (XRD) analysis confirmed the monoclinic structure of the samples with the lattice parameters and unit cell volume fitting well to the general trends of the (Ga_1−x_In_x_)_2_O_3_ solid solution series. Broad emission spectra ranging from 1.5 to 3.5 eV were registered for all samples. Luminescence spectra showed violet, blue, and green emission elementary bands. The luminescence intensity was found to vary depending on the synthesis atmosphere. An argon synthesis atmosphere leads to increasing violet luminescence and decreasing green luminescence. Intense bands at about 4.5 and 5.0 eV and a low-intensity band at 3.3 eV are presented in the excitation spectra. The electrical conductivity of the samples was also determined depending on the synthesis atmosphere. The high-resistance samples obtained in an oxygen atmosphere exhibited activation energy of around 0.98 eV. Samples synthesized in an argon atmosphere demonstrated several orders of magnitude higher conductivity with an activation energy of 0.15 eV. The results suggest that the synthesis atmosphere is crucial in determining the luminescent and electrical properties of undoped β-Ga_2_O_3_-In_2_O_3_ solid solution samples, offering the potential for various optoelectronic applications.

## 1. Introduction

In recent years, there has been a growing interest in exploring novel semiconductor materials that possess improved properties and expand the potential of application in various technological and scientific areas. Among these materials, a monoclinic gallium oxide (β-Ga_2_O_3_) has attracted significant attention due to its unique physics and technical characteristics. Gallium oxide is a well-known wide-bandgap semiconductor with remarkable structural, electronic, optical, luminescent, and thermal properties, making it attractive for applications in power electronics, optoelectronics, solar applications, sensor technologies, etc. [1,2,3,4,5,6]. It exhibits several structural polymorphs including the corundum-like (α), monoclinic (β), defective spinel (γ), and two variations of orthorhombic structure (ε and δ) [7]. Among these, the monoclinic phase (β-Ga_2_O_3_) stands out as the most common, being both the most stable and the easiest to obtain under ambient conditions [8,9,10,11,12].

Solid solutions of monoclinic gallium oxide with aluminum or indium oxide is a well-known approach for tuning the physical properties of β-Ga_2_O_3_ over a wide range [13,14,15,16]. This tuning is achieved primarily by modifying the crystal structure and electronic band structure of the material (see [11,13,17] and references therein). Several recent studies confirm the high effectiveness of this approach for the enhancement of functional properties of β-Ga_2_O_3_-based semiconductor devices [18,19,20,21].

Solid solutions based on gallium oxide exhibit good optical properties, including high transparency in the visible and ultraviolet spectral regions. The unique combination of luminescent and electrical properties in β-Ga_2_O_3_-In_2_O_3_ solid solutions opens new possibilities for energy-efficient lighting. The transparency and tunable wide bandgap make them potential candidates for optoelectronic devices, such as photodetectors and light-emitting diodes (LEDs) [4,6]. The surface properties of these solid solutions can be adapted to enhance their sensitivity for gas molecules, enabling the development of selective and sensitive gas sensors [3].

As it is known, doping of β-Ga_2_O_3_ gallium oxide with a divalent Mg impurity makes it possible to obtain a high-resistance material [1,22], which is necessary for the manufacture of power diodes and transistors based on Schottky barriers. At the same time, the ability to change the band gap when forming β-Ga_2_O_3_-In_2_O_3_ solid solutions opens up additional opportunities for creating heterostructures based on these wide-gap semiconductor materials. However, the influence of the divalent Mg impurity on the electrical, optical, and luminescent properties of the β-Ga_2_O_3_-In_2_O_3_ solid solution has not yet been studied.

The technological conditions of the production of gallium oxide and their solid solutions, in particular the atmosphere of synthesis, have a decisive influence on the formation of intrinsic point defects in the material and in such a way have a strong impact on their physical properties. Therefore, this study aims to investigate in detail the crystal structure, photoluminescent properties and electrical conductivity of the β-(Ga_1−x_In_x_)_2_O_3_ solid solutions synthesized under oxygen or an inert gas (argon) atmosphere. The study was performed both for nominally pure and Mg^2+^-doped β-(Ga_1−x_In_x_)_2_O_3_ solid solutions synthesized under different gas atmospheres. This approach allowed for a comprehensive analysis of how the synthesis atmosphere affects the material properties.

## 2. Experimental Details

Polycrystalline undoped β-(Ga_1−x_In_x_)_2_O_3_ solid solution was obtained using the high-temperature solid-state chemical reaction method. The starting materials for this synthesis were gallium oxide (β-Ga_2_O_3_) and indium oxide (In_2_O_3_) with a minimum purity grade of 4N. The initial mixture of solid solution components contained 20% indium oxide (β-(Ga_0.8_In_0.2_)_2_O_3_). Some β-(Ga_0.8_In_0.2_)_2_O_3_ samples were synthesized in a pure oxygen atmosphere at approximately 1 atm pressure, while others were synthesized under low oxygen partial pressure (~0.01 atm) conditions using an inert argon gas atmosphere.

The oxide powder mixture was initially mechanically blended in an agate mortar until a homogeneous mass was achieved. The synthesis samples were obtained as flat tablets with a diameter of 8 mm and a thickness of 1 mm through mechanical pressing. Subsequently, the tablets were wrapped in platinum foil and placed in quartz ampoules. These ampoules and the samples were placed inside an electric furnace and synthesized at 1300 °C for 12 h. Before the synthesis process, the air was removed from the ampoule, and the volume was filled with the appropriate gas by connecting a cylinder with oxygen or argon. Undoped β-(Ga_0.8_In_0.2_)_2_O_3_ ceramic samples synthesized in an oxygen atmosphere exhibited a white color, while those synthesized in an argon atmosphere were of a bluish color.

XRD studies were performed to analyze the structure of the synthesized solid solution samples. The materials were characterized using an Aeris benchtop powder diffractometer (Malvern Panalytical, Worcestershire, UK) equipped with a PIXcel^1D^ strip detector. Experimental diffraction data were collected within a 2θ range of 10 to 105 degrees, with a 2θ step of 0.01°, using filtered Cu Kα radiation (λ = 1.54185 Å). To determine the precise values of the structural parameters, the experimental XRD patterns were subjected to full-profile Rietveld refinement using the WinCSD software package with Version 4.19 [23]. This refinement procedure involved refining the unit cell dimensions, positional and displacement parameters of atoms, profile parameters, texture and corrections for absorption and instrumental sample shift. Occupancies of atomic sites were refined in a soft mode during the final refinement stage.

The photoluminescence (PL) and photoluminescence excitation (PLE) spectra were studied at room temperature using a Solar CM2203 spectrofluorometer. A Hamamatsu R928 type photomultiplier with a spectral resolution of 1 nm was used to record the spectra. The PL spectra were corrected for a spectral response of the system. The PLE spectra were corrected for the xenon lamp emission spectrum.

The electrical conductivity of the studied polycrystalline samples was determined using the conventional two-probe method. Indium contacts were applied to the tablet samples on both (front and back) surfaces. Current–voltage characteristics measurements indicated that indium formed reliable ohmic contacts with high-resistance samples. The currents measured by an electrometer ranged from 10^−3^ to 10^−14^ A.

## 3. Results

### 3.1. Phase Composition and Crystal Structure Parameters

**Figure 1:**
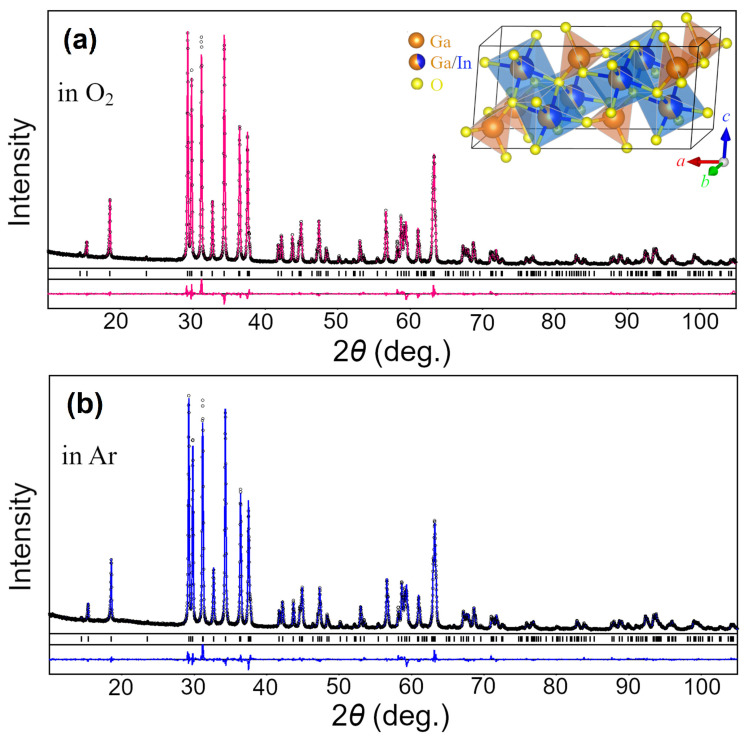
Graphical results of Rietveld refinement of the structure of (Ga_1−x_In_x_)_2_O_3_ samples synthesized in oxygen (**a**) and argon (**b**) atmospheres. Experimental XRD patterns (small black circles) are shown in comparison with a calculated pattern for β-Ga_2_O_3_ structure (pink and blue lines). Short vertical bars indicate the positions of Bragg’s maxima in the β-Ga_2_O_3_ structure. The inset shows polyhedral representations of the structure showing mixed occupancy of octahedra with Ga and In atoms in (Ga_1−x_In_x_)_2_O_3_ samples.

XRD examination revealed that as-obtained samples with nominal composition (Ga_0.8_In_0.2_)_2_O_3_ adopt a monoclinic structure isotypic with β-Ga_2_O_3_. No traces of impurity phase(s) were detected in the samples, being further proved by full profile Rietveld refinement performed in space group *C*2/*m*. As a starting model, the atomic coordinates in β-Ga_2_O_3_ structure derived from X-ray single crystal data [24] and standardized according to Pearson’s Crystal Data were used. Graphical results of Rietveld refinement presented in Figure 1 demonstrate an excellent agreement between experimental and calculated XRD patterns for the materials annealed either in oxygen or argon atmospheres. Refined values of the lattice parameters, fractional coordinates of atoms and their displacement parameters are collected in Table 1. It was found that the indium atoms in the (Ga_1−x_In_x_)_2_O_3_ structure substitute for Ga2 atoms in octahedral positions, whereas the tetrahedral Ga1 sites are occupied solely with Ga species (see Table 1). For the first time, a supposition that In resides on the octahedral sites of β-Ga_2_O_3_ structure was made in the pioneering work of Shannon and Prewitt more than 55 years ago [25]. Later on, it was validated by perturbed angular correlation measurements [26] and confirmed by an analysis of XRD data [27,28]. Finally, the exclusive preference of In^3+^ ions for octahedral sites was recently proved by comprehensive structural investigations of (Ga_1−x_In_x_)_2_O_3_ powders doped with Cr^3+^ [29] and co-doped with Cr^3+^/Ca^2+^ ions [17].

**Figure 2:**
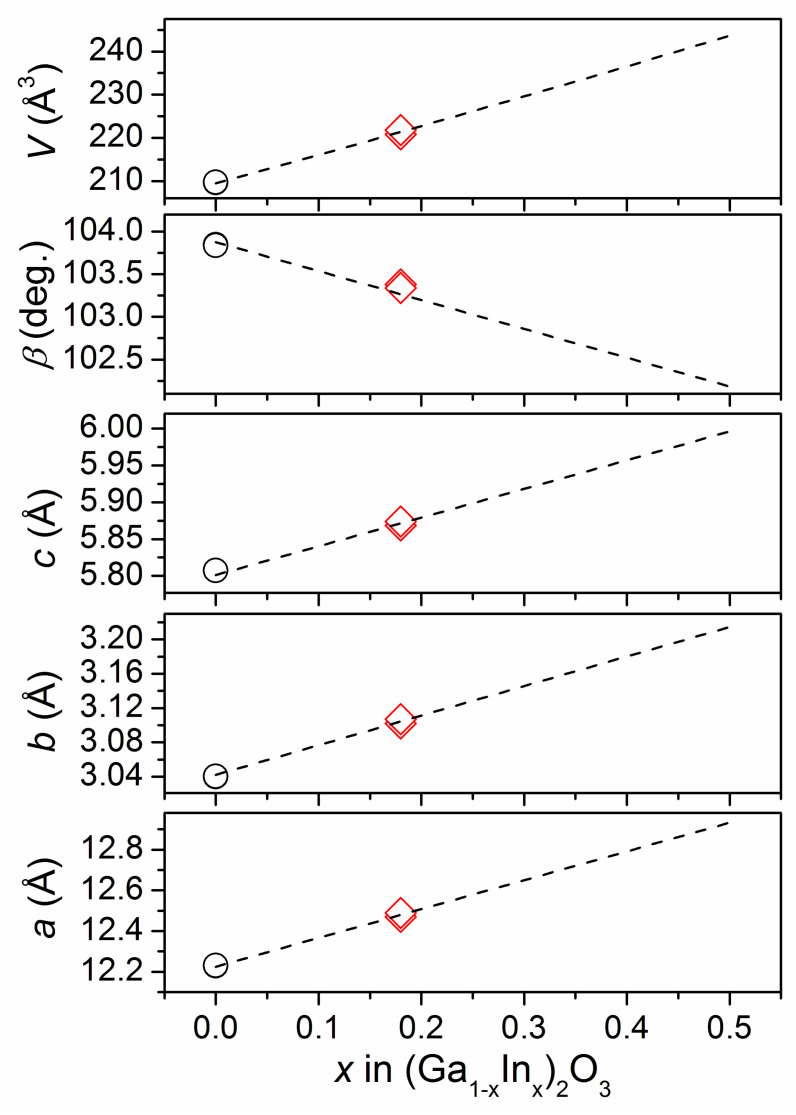
Evolution of the lattice parameters and unit cell volume vs. indium content in (Ga_1−x_In_x_)_2_O_3_ solid solution. Dashed lines represent linear fits performed by [11] for five data sets for the powder and single crystal (Ga_1−x_In_x_)_2_O_3_ materials taken from Refs. [25,27,30,31,32]. Unit cell dimensions of the nominally pure β-Ga_2_O_3_ [33] and two (Ga_1−x_In_x_)_2_O_3_ samples studied in the present work are shown by circles and red diamonds, respectively.

From the refined site occupancies, the composition of the samples annealed in O_2_ and Ar atmospheres can be calculated as Ga_1.625_In_0.375_O_3_ (Ga_0.813_In_0.187_)_2_O_3_ and Ga_1.645_In_0.355_O_3_ (Ga_0.823_In_0.177_)_2_O_3_, respectively, which are close to the nominal starting formulae (Ga_0.8_In_0.2_)_2_O_3._ Similar sample composition can be evaluated by comparing their unit cell dimensions with the literature data for β-(Ga_1−x_In_x_)_2_O_3_ solid solution series, from which the In content (x) in the samples under present investigation can be estimated as 0.18 (Figure 2).

Comparative analysis of the refined structural data shows that the lattice parameters and unit cell volume of the sample synthesized in the argon atmosphere are detectably higher than those of the oxygen-treated sample. In addition, a detectable difference in the displacement parameters (*B*_iso_) of oxygen atoms in both structures is observed (see Table 1). Both these observations point to the change in the defect structure and the creation of extra oxygen vacancies after heat treatment of the material in the Ar atmosphere.

Based on the refined values of the lattice parameters and atomic coordinates, the nearest interatomic distances in the (Ga_1−x_In_x_)_2_O_3_ samples annealed in O_2_ and Ar atmospheres were calculated (see Table 2). The average metal–oxygen distances inside (Ga/In)O_6_ octahedra in both (Ga_1−x_In_x_)_2_O_3_ structures are considerably higher compared with corresponding values for the parent β-Ga_2_O_3_ structure, whereas the average distances inside GaO_4_ tetrahedra remains practically unchanged. This observation additionally proves our conclusion on the substitution of In ions solely in octahedral positions of β-Ga_2_O_3_ structure.

### 3.2. Photoluminescent Properties

**Figure 3:**
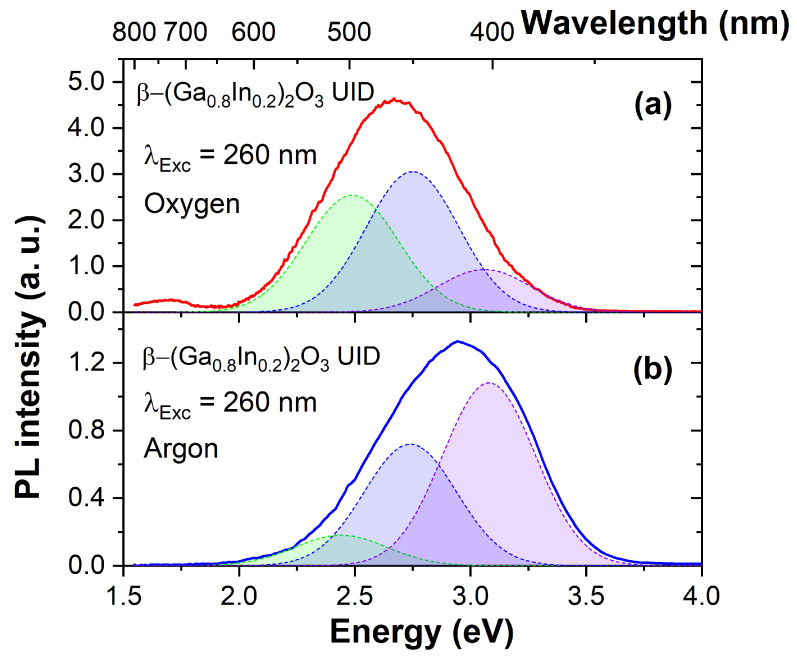
Photoluminescence spectra of β-(Ga_0.8_In_0.2_)_2_O_3_ ceramics synthesized in oxygen (**a**) and argon (**b**) atmospheres. The elementary Gaussian peaks represent green, blue, and violet emission bands.

Luminescence spectra obtained for β-(Ga_0.8_In_0.2_)_2_O_3_ ceramic samples when excited by the UV light at 260 nm near the fundamental absorption edge are shown in Figure 3. When synthesized in an oxygen atmosphere (Figure 3a), the photoluminescence exhibited a broad spectrum from 1.5 to 3.5 eV, with the emission maximum around 2.63 eV. This complex luminescence spectrum can be deconvoluted into elementary Gaussian peaks, with maxima in the violet (3.08 eV), blue (2.74 eV), and green (2.47 eV) spectral regions. Each elementary luminescence band has a full width at half maximum (FWHM) of approximately 0.4 eV. Notably, the blue and green luminescence bands were the most prominent in ceramics syntheses in oxygen.

In the case of the ceramics synthesized in an argon atmosphere (Figure 3b), a decrease in the overall luminescence intensity was observed, along with a shift of the emission maximum (~2.9 eV) towards shorter wavelengths. Consequently, reducing the oxygen partial pressure in the synthesis atmosphere leads to an increased intensity of the short-wavelength violet luminescence band and a decreased intensity of the green luminescence band.

**Figure 4:**
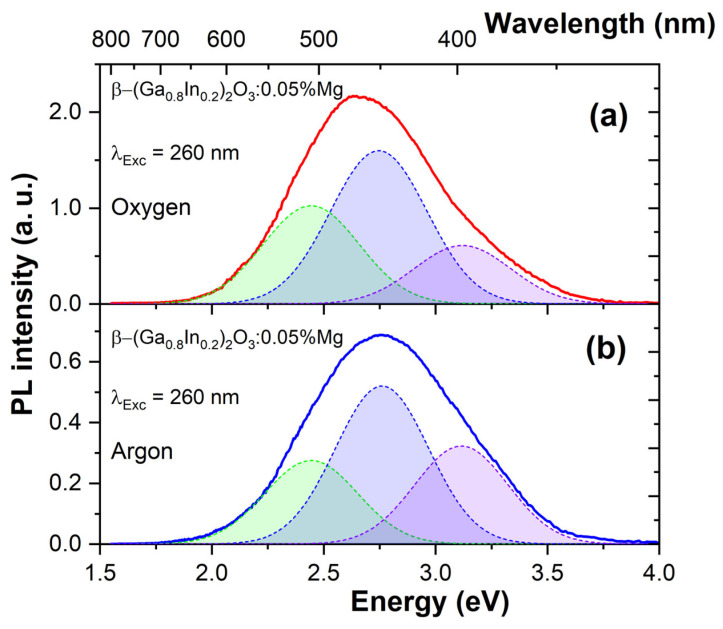
Photoluminescence spectra of β-(Ga_0.8_In_0.2_)_2_O_3_:0.05%Mg ceramics synthesized in oxygen (**a**) and argon (**b**) atmospheres. The elementary Gaussian peaks represent green, blue, and violet emission bands.

Doping with Mg^2+^ (Figure 4) decreased the integrated luminous intensity by approximately two times compared to undoped ceramics. The luminescence maximum for β-(Ga_0.8_In_0.2_)_2_O_3_:Mg samples synthesized in an oxygen atmosphere is about 2.67 eV. The broad emission band of the β-(Ga_0.8_In_0.2_)_2_O_3_:Mg ceramic was also decomposed into the same elementary Gaussian curves. The β-(Ga_0.8_In_0.2_)_2_O_3_:Mg ceramic synthesized in argon (Figure 4b) has a maximum emission at 2.7 eV (459 nm). Synthesis in an argon atmosphere also increased the relative intensity of the blue and violet luminescence bands.

**Figure 5:**
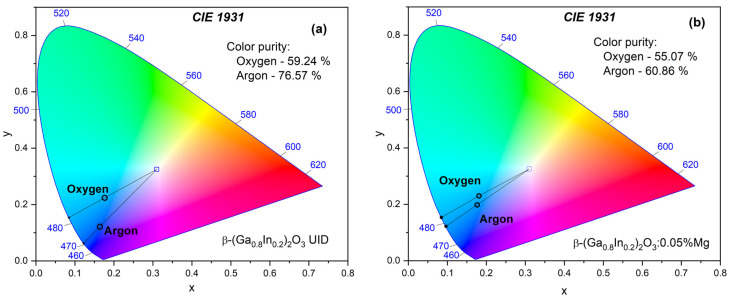
CIE 1931 chromaticity diagram for β-(Ga_0.8_In_0.2_)_2_O_3_ (**a**) and β-(Ga_0.8_In_0.2_)_2_O_3_:0.05%Mg (**b**) ceramics synthesized in oxygen and argon atmospheres.

Based on the PL spectra for the two types of synthesized materials, the CIE 1931 chromaticity coordinates were estimated (see Figure 5). These coordinates are typically plotted relative to the CIE standard illuminant D65 (natural white daylight), which has coordinates (x_i_ = 0.3127, y_i_ = 0.3290) that allow for calculating the color purity (CP).

For β-(Ga_0.8_In_0.2_)_2_O_3_ solid solution samples synthesized in oxygen, the chromaticity coordinates were x = 0.1761 and y = 0.2241, which might appear bluish-green or turquoise. The dominant wavelength coordinates for this sample were x_d_ = 0.0846 and y_d_ = 0.1537, corresponding to the wavelength 482 nm. On the other hand, for samples synthesized in an argon atmosphere, the chromaticity coordinates were x = 0.1639 and y = 0.1214, placing the color at a different point, which appears as a saturated blue. Also, the dominant wavelength coordinates for this sample were x_d_ = 0.1223 and y_d_ = 0.0625, pointing at 470 nm (Figure 5a). For samples of a solid solution doped with Mg^2+^ ions synthesized in oxygen, the color coordinates were x = 0.085 and y = 0.154, and for samples synthesized in an argon atmosphere, x = 0.096 and y = 0.122. The dominant coordinates of wavelengths for these samples corresponded to emission wavelengths of 483 and 476 nm, respectively (Figure 5b).

The CP calculation, which indicates the color saturation or intensity relative to the specified standard illuminant (x_i_, y_i_) and dominant wavelength coordinates (x_d_, y_d_), was established for both samples. As follows, the CP for the sample β-(Ga_0.8_In_0.2_)_2_O_3_ synthesized in an argon atmosphere was relatively high, near 76.6%, while, for the sample synthesized in oxygen, CP was estimated as 59.2%. The CP for the β-(Ga_0.8_In_0.2_)_2_O_3_:Mg sample was slightly lower and amounted to 60.86% and 55.07% for the samples synthesized in argon and oxygen atmospheres, respectively.

**Figure 6:**
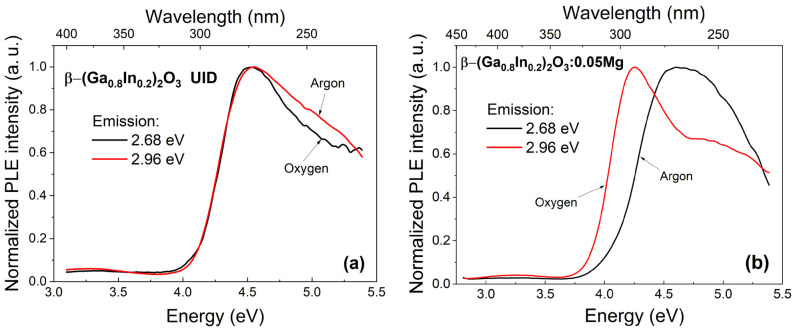
The photoluminescence excitation spectra of the β-(Ga_0.8_In_0.2_)_2_O_3_ (**a**) and β-(Ga_0.8_In_0.2_)_2_O_3_:0.05%Mg (**b**) ceramics synthesized in oxygen (black curve) and argon (red curve) atmospheres.

The typical PLE spectra for β-(Ga_0.8_In_0.2_)_2_O_3_ and β-(Ga_0.8_In_0.2_)_2_O_3_:Mg solid solutions synthesized either in oxygen or argon atmospheres are shown in Figure 6. These spectra are similar in the energy range of 3.0–5.5 eV due to the overlap of the elementary luminescence bands. Moreover, the excitation spectra of all elementary emission bands reach their maximum intensity at energies near ~4.5 eV for β-(Ga_0.8_In_0.2_)_2_O_3_.

The excitation spectra overlap with the material’s fundamental absorption region of 4.7–5.5 eV and the transparency region of 2.7–4.5 eV. The low-intensity band at 3.3 eV, located in the transparency region of the material, has an excitation intensity of approximately 0.1 from the maximum intensity. It should be noted that these broad luminescence excitation bands correlate with the positions of the fundamental absorption edge and photoconductivity maxima in β-(Ga_0.8_In_0.2_)_2_O_3_ solid solution single crystals [27]. For β-(Ga_0.8_In_0.2_)_2_O_3_ solid solution samples synthesized in an argon atmosphere (Figure 5), a slight increase in the relative intensity of the excitation bands at about 3.3 and above 4.5 eV was observed.

For the β-(Ga_0.8_In_0.2_)_2_O_3_:Mg samples synthesized in an oxygen atmosphere (Figure 6b), a stronger change in the shape of the luminescence excitation spectrum is observed. The maximum of the luminescence excitation curve is in the longer wavelength region of the spectrum. The displacement of the maximum of the integral excitation curve occurred due to a substantial increase in the intensity of the 4.2 eV band, which becomes the main luminescence excitation band.

### 3.3. Electrical Conductivity

**Figure 7:**
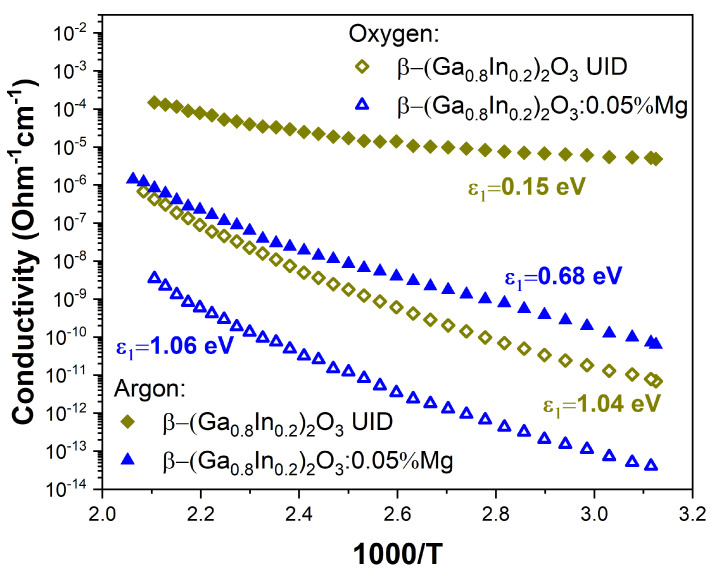
The temperature dependence of the electrical conductivity of β-(Ga_0.8_In_0.2_)_2_O_3_ and β-(Ga_0.8_In_0.2_)_2_O_3_:0.05%Mg solid solutions synthesized in oxygen or argon gas atmospheres.

Figure 7 shows the temperature dependence of the electrical conductivity of undoped and Mg-doped β-(Ga_0.8_In_0.2_)_2_O_3_ solid solution samples. The electrical conductivity of UID and Mg-doped β-(Ga_0.8_In_0.2_)_2_O_3_ ceramics, which were synthesized in an oxygen atmosphere, was relatively low at 295 K. It varied in the range from ~1.2∙10^−13^ Ohm^−1^∙cm^−1^ for samples doped with Mg^2+^ to 5∙10^−12^ Ohm^−1^∙cm^−1^ for UID samples. The activation energy of electrical conductivity in such high-resistance samples was significant and was in the energy range of 1.04–1.06 eV.

At the same time, for β-(Ga_0.8_In_0.2_)_2_O_3_ ceramics samples that were synthesized in an argon atmosphere, the conductivity was much higher and varied greatly depending on the doping impurity from ~1.3 × 10^−10^ Ohm^−1^∙cm^−1^ for samples doped with Mg^2+^ to ~1.1 × 10^−5^ Ohm^−1^∙cm^−1^ for UID samples. The activation energy of electrical conductivity in such samples was 0.15 and 0.68 eV for UID and Mg-doped β-(Ga_0.8_In_0.2_)_2_O_3_ samples. Note that in all cases, the electrical conductivity of β-(Ga_0.8_In_0.2_)_2_O_3_ samples synthesized in an argon atmosphere was higher than that of samples synthesized in an oxygen atmosphere.

## 4. Discussion

The luminescence spectra of polycrystalline samples of β-(Ga_0.8_In_0.2_)_2_O_3_ (shown in Figure 3) resemble the luminescence of pure gallium oxide reported elsewhere [34,35,36,37,38,39]. Doping with divalent Mg^2+^ metals did not lead to significant changes in the form of the luminescence spectrum. Results of the present work suggest that relative intensities of the elementary luminescence bands are considerably redistributed depending on the synthesis atmosphere applied. When synthesized in an oxygen atmosphere, the relative intensity of long-wave luminescence increases. Conversely, synthesis in an atmosphere of inert argon gas increases the intensity of short-wavelength luminescence bands.

The luminescence of β-(Ga_0.8_In_0.2_)_2_O_3_ solid solutions can be explained using the models of luminescence centers proposed previously for gallium oxide [34,35,36,37,38,39]. In particular, the violet, blue, and green luminescence occurs due to the radiative recombination of carriers through DAP (donor–acceptor pairs). However, the elementary luminescence bands in β-(Ga_0.8_In_0.2_)_2_O_3_ are shifted towards longer wavelengths with respect to β-Ga_2_O_3_, which is consistent with a decrease in the bandgap of β-Ga_2_O_3_ when alloyed with In_2_O_3_. Like gallium oxide, β-(Ga_0.8_In_0.2_)_2_O_3_ contains background donor impurities (e.g., Si^4+^, Ge^4+^, Sn^4+^) at a relatively high concentration of around 2–10 ppm. Shallow donors, such as background impurities of tetravalent metals, interstitial gallium (Ga_i_), or deep donors, such as oxygen vacancies with two trapped electrons (V_O_^2+^ + 2e), can act as donor components of DAP. The acceptor component of DAP consists of native acceptor-type defects, such as gallium vacancies (V_Ga_^3−^) and bi-vacancies (V_Ga_^3−^-V_O_^2+^) [40,41]. The β-(Ga_0.8_In_0.2_)_2_O_3_ contains both shallow and deep donors, allowing the DAP luminescence bands to appear in a wide range of wavelengths from 350 to 500 nm. For example, by the authors [34], the blue luminescence band has been related to the transitions between deep donors such as oxygen vacancies (V_O_) or interstitial Ga (Ga_i_) and deep acceptors such as Ga vacancies (V_Ga_) or V_O_-V_Ga_ complexes. Violet luminescence in β-(Ga_0.8_In_0.2_)_2_O_3_ can also occur by recombining electrons with self-trapped holes or holes localized on defects [42]. Further studies with a controlled change in the concentration of defects that form the donor and acceptor levels are required for an unambiguous correlation of emission bands with host point defects and impurities.

During synthesis in an oxygen atmosphere, the high partial pressure of oxygen increases the energy required to form oxygen vacancies, decreasing their concentration in such a way. A low partial pressure of oxygen, vice versa, reduces the energy needed to form oxygen vacancies and increases the energy required to create gallium vacancies. Therefore, oxygen vacancies become the dominant defects in the ceramics synthesized in the argon atmosphere. As it was shown above, β-(Ga_0.8_In_0.2_)_2_O_3_ has a monoclinic structure similar to β-Ga_2_O_3_, where 40% of gallium atoms in octahedral coordination are replaced by indium atoms (see also Refs. [17,25]). Therefore, similar energy dependencies for forming intrinsic defects should be observed in both β-Ga_2_O_3_ and β-(Ga_0.8_In_0.2_)_2_O_3_.

The low conductivity of β-(Ga_0.8_In_0.2_)_2_O_3_ samples synthesized in an oxygen atmosphere suggests that the total concentration of compensating acceptors is comparable to that of donors. The raw material specification indicates a lower concentration of background impurity of acceptors than donors. Consequently, samples synthesized in an oxygen atmosphere have more native acceptors such as gallium vacancies or bi-vacancies, and their concentration is consistently higher in samples synthesized in oxygen than in those synthesized in argon.

The acceptors generate energy levels near the top of the valence band, and transitions from electron-filled acceptor levels to the conduction band give rise to excitation bands. The excitation band around 4.5 eV may be associated with electronic transitions from native acceptor defect levels to the conduction band. All undoped solid solution samples exhibit an excitation band with a peak near 3.3 eV. This band can be attributed to defects that form during synthesis in an argon atmosphere with a low partial pressure of oxygen, such as oxygen vacancies. Specifically, it may arise when an electron is excited from an oxygen vacancy to the conduction zone. However, further research is required to confirm this hypothesis.

Divalent Mg^2+^ ions replace Ga^3+^ ions in the crystal lattice. When doping with Mg^2+^ impurities, a sufficiently high concentration of positively charged oxygen vacancies V_O_^2+^ is also formed to ensure the electrical neutrality of the material. A high concentration of V_O_^2+^, in turn, leads to an increase in the probability of the formation of divacancies. The Mg^2+^ ions create acceptor levels near the top of the valence band. Transitions of electrons from the energy levels of compensating acceptors to the conduction zone are manifested in the excitation bands. The β-(Ga_0.8_In_0.2_)_2_O_3_:Mg^2+^ samples synthesized in an oxygen atmosphere always had very high resistance. In such samples, the concentration of intrinsic and impurity background donors (Si^4+^, Sn^4+^, etc.) is negligible. Therefore, only a part of the acceptors participates in the compensation, while band–band transitions dominate the excitation spectrum.

The β-(Ga_0.8_In_0.2_)_2_O_3_:Mg^2+^ samples synthesized at low oxygen pressure have a conductivity of ~10^−10^ Ohm^−1^∙cm^−1^. The concentration of impurity Mg^2+^ acceptors is high and exceeds the concentration of intrinsic acceptors (V_Ga_^3−^ or V_Ga_^3−^-V_O_^2+^); therefore, the conductivity increased primarily due to additional donors formed during synthesis under conditions of low oxygen partial pressure. Since the donor concentration increased, more acceptors can capture electrons. Since the concentration of the Mg^2+^ dopant is higher than the concentration of its native acceptor defects, such as V_Ga_^3−^ or V_Ga_^3−^-V_O_^2+^, the intensity of the impurity excitation band is also higher. Transitions of electrons from Mg^2+^ acceptor levels to the conduction band appear as an intense dominant excitation band located in the transparency region before the fundamental absorption edge at energies of 4.2 eV (295 nm) for β-(Ga_0.8_In_0.2_)_2_O_3_:Mg.

## 5. Conclusions

The synthesis of undoped and Mg^2+^-doped β-(Ga_1−x_In_x_)_2_O_3_ solid solutions (x = 0.2) in different atmospheres allowed us to investigate the effect of the oxygen partial pressure on native point defects responsible for the material’s properties. The XRD analysis confirmed the desired monoclinic structure of the samples without any impurity phases. It was revealed that In atoms in the (Ga_1−x_In_x_)_2_O_3_ structure substitute for Ga2 atoms in octahedral positions, whereas the tetrahedral Ga1 sites are occupied solely with Ga species. Moreover, the XRD data suggest the creation of extra oxygen vacancies in the material synthesized in the argon atmosphere.

The luminescence spectra of the materials synthesized either in an oxygen or argon atmosphere exhibited the same broad emission bands in the violet, blue, and green regions; however, their relative intensities were revealed to be quite different. In particular, the synthesis in an oxygen atmosphere led to enhanced long-wavelength luminescence and lower electrical conductivity, likely due to decreased oxygen vacancies and increased gallium vacancies. In contrast, synthesis in an argon atmosphere increased short-wavelength luminescence and electrical conductivity, suggesting a higher concentration of oxygen vacancies. The observed luminescence behavior in the studied β-(Ga_0.8_In_0.2_)_2_O_3_ solid solutions can be explained by the radiative recombination of carriers through the donor-acceptor pairs (DAP) like in pristine β-Ga_2_O_3_. The conductivity of samples synthesized in an argon atmosphere is six orders of magnitude higher than those synthesized in an oxygen atmosphere. The main excitation band at about 5.0 eV also confirms the involvement of interband transitions in the luminescent behaviour of the materials.

This study highlights the importance of controlling the synthesis atmosphere when fabricating β-Ga_2_O_3_-based solid solutions. The results provide valuable information about the relationship between native defects, luminescent properties, and electrical conductivity, which are crucial for understanding the optoelectronic behavior of these materials. The relatively high color purity of the β-(Ga_0.8_In_0.2_)_2_O_3_ samples synthesized in an argon atmosphere, together with their high conductivity and intense luminescence, indicate that these materials are promising for use as blue light sources.

## Figures and Tables

**Table 1:** Lattice parameters, coordinates, and displacement parameters of atoms in monoclinic structures of β-(Ga_1−x_In_x_)_2_O_3_ annealed in O_2_ and Ar atmospheres (SG *C*2/*m*, *Z* = 4).

Lattice Parameters	Atoms, Sites	*x/a*	*y/b*	*z/c*	*B_iso/eq_,* Å^2^	Occupancy
β-(Ga_1−x_In_x_)_2_O_3_ in O_2_; *R*_I_ = 0.0381, *R*_P_ = 0.0713						
*a* = 12.4697(2) Å	Ga1, 4*i*	0.09048(10)	0	0.2929(3)	0.82(3)	1.01(1) Ga^3+^
*b* = 3.10206(5) Å	Ga2, 4*i*	0.34367(7)	0	0.1885(2)	0.93(3)	0.63(2) Ga^3+^ + 0.38(2) In^3+^
*c* = 5.86855(9) Å	O1, 4*i*	0.1628(4)	0	0.6187(11)	1.1(2)	O^2−^
*β* = 103.379(1) o	O2, 4*i*	0.1714(4)	0	0.0653(14)	1.6(2)	O^2−^
*V* = 220.85(1) Å3	O3, 4*i*	0.5123(4)	0	0.2460(8)	0.7(2)	O^2−^
Texture axis and parameter: [1 0 0] 0.563(5)						
β-(Ga_1−x_In_x_)_2_O_3_ in Ar; *R*_I_ = 0.0454, *R*_P_ = 0.0733						
*a* = 12.4886(2) Å	Ga1, 4*i*	0.09041(10)	0	0.2926(3)	0.76(3)	1.00(1) Ga^3+^
*b* = 3.10717(6) Å	Ga2, 4*i*	0.34347(7)	0	0.1884(2)	0.78(3)	0.65(2) Ga^3+^ + 0.35(2) In^3+^
*c* = 5.87368(10) Å	O1, 4*i*	0.1629(4)	0	0.6191(12)	1.9(2)	O^2−^
*β* = 103.337(1) o	O2, 4*i*	0.1707(4)	0	0.0658(13)	1.0(2)	O^2−^
*V* = 221.78(1) Å3	O3, 4*i*	0.5137(4)	0	0.2487(9)	1.6(2)	O^2−^
Texture axis and parameter: [1 0 0] 0.584(5)						

**Table 2:** Individual and average interatomic distances (in Å) inside GaO_4_ tetrahedra and *M*_o_O_6_ octahedra in two (Ga_1−x_In_x_)_2_O_3_ materials annealed in O_2_ and Ar atmospheres in comparison with the parent Ga_2_O_3_ structure. Besides individual distances, the average distances inside polyhedra and their increment are presented as well. *M* denote the mixture of Ga/In cations in octahedral positions.

Atoms	Ga_2_O_3_ Ref. [24]	(Ga_1−x_In_x_)_2_O_3_		Atoms	Ga_2_O_3_ Ref. [24]	(Ga_1−x_In_x_)_2_O_3_	
		in O_2_	in Ar			in O_2_	in Ar
Ga-O3 (×2)	*1.832*	1.819(3)	1.812(3)	*M*-O1 (×2)	*1.937*	1.933(4)	1.934(4)
Ga-O2	*1.863*	1.850(7)	1.842(7)	*M*-O3	*1.936*	2.051(5)	2.073(5)
Ga-O1	*1.835*	1.916(7)	1.923(7)	*M*-O2	*2.005*	2.102(6)	2.112(6)
				*M*-O2 (×2)	*2.074*	2.128(5)	2.134(5)
Ga-O_4_(ave), *increment*	*1.841*	1.851 *+0.54%*	1.847 +0.33%	*M*O_6_(ave), *increment*	*1.994*	2.046 *+2.61%*	2.054 *+3.00%*

## Data Availability

The data of this study are available on request to the corresponding author.
